# Associations between executive functions, intelligence and adaptive behaviour in children and adolescents with mild intellectual disability

**DOI:** 10.1177/17446295221095951

**Published:** 2022-05-11

**Authors:** Sissel Gravråkmo, Alexander Olsen, Stian Lydersen, Jo Magne Ingul, Lucy Henry, Merete G Øie

**Affiliations:** Regional Centre for Habilitation at the Regional Centre for Child and Youth Mental Health and Child Welfare, Department of Mental Health, Faculty of Medicine and Health Sciences, 8018 NTNU - Norwegian University of Science and Technology, Trondheim, Norway; Department of Habilitation Services at the Children´s Clinic, St. Olav’s Hospital, Trondheim University Hospital, Trondheim, Norway; Department of Psychology, 8018 Norwegian University of Science and Technology, Trondheim, Norway; Department of Physical Medicine and Rehabilitation, St. Olav’s Hospital, Trondheim University Hospital, Trondheim, Norway; Regional Centre for Child and Youth Mental Health and Child Welfare, Department of Mental Health, Faculty of Medicine and Health Sciences, 8018 NTNU – Norwegian University of Science and Technology, Trondheim, Norway; Division of Language and Communication Science, City, 4895 University of London, UK; Department of Psychology, 6305 University of Oslo, Oslo, Norway

**Keywords:** Adaptive behaviour, developmental disabilities, executive function, intellectual disability, neurodevelopmental disorders

## Abstract

**Background:** The purpose of this study was to explore the role of everyday executive functions in relation to intelligence and adaptive behaviour in children and adolescents with mild intellectual disability. **Method:** A group of children and adolescents, previously diagnosed with mild intellectual disability were assessed according to intelligence, everyday executive functions and adaptive behaviour. The association between everyday executive functions and intelligence was examined, and it was explored whether intelligence or everyday executive functions would best predict adaptive behaviour. **Results:** Adaptive behaviour was significantly predicted by executive functions, but not by intelligence. Nor was intelligence significantly related to everyday executive functions in this group. **Conclusions:** Although fundamental in diagnosing intellectual disability, intelligence cannot predict adaptive behaviour. Assessing everyday executive functions and adaptive behaviour, as well as acknowledging the strong association between them, provides valuable information in the process of optimizing developmental support for children and adolescents with mild intellectual disability.

## Introduction

Since the 1950s, more emphasis has gradually been placed on adaptive behaviour in the diagnosis and understanding of intellectual disability (Tassé et al., 2016). A model of intelligence and adaptive behaviour as separate, but related constructs has been supported (Keith et al., 1987), and the importance of assessing both constructs and weighing them equally in the process of diagnosing intellectual disability has been emphasized (Tassé et al., 2016). It has been suggested that the ICD-10 (World Health Organization, 2016) has not been sufficiently distinct on the requirement of concurrent impaired adaptive functioning in the diagnosis of intellectual disability, which has been adjusted in the ICD-11 (RHABU, 2019; World Health Organization, 2021). In the DSM-5, the requirement of significant impairment in both intelligence and adaptive behaviour is explicit (American Psychiatric Association, 2013), nevertheless, the risk of thinking errors related to the ordering of criteria, and possible misconceptions regarding requirement of causality between criteria have been debated (Tassé et al., 2016). From the 1980s, a rapidly growing body of research concerning executive functions has emerged, but the significance of executive functions, and how they relate to intelligence and adaptive behaviour in children and adolescents with mild intellectual disability has received less attention. The purpose of the current study was to examine the association between intelligence and executive functions, and to explore whether intelligence or executive functions was the best predictor of adaptive behaviour in children and adolescents with mild intellectual disability.

Adaptive behaviour has over the years become an increasingly well-defined construct, encompassing social, communication and practical skills that can be empirically assessed using validated inventories (Tassé et al., 2012). One example of an adaptive functioning scale that is widely used is the Vineland Adaptive Behaviour Scales (Sparrow et al., 2005). It has been proposed that behavioural assessments of intellectual abilities fail to discover the adaptive resources, and tend to place too much emphasis on the burden (Maulik et al., 2011). As for the relation between intelligence and adaptive behaviour, findings suggest that only communication skills, from the three main domains of adaptive behaviour (communication, daily living skills and socialization), are linked to intelligence (Di Nuovo and Buono, 2011). A recent Meta-Analysis reported varying magnitudes of correlation between intelligence and adaptive behaviour across intellectual levels, with positive correlations for IQ (intelligence quotient) scores between 50 and 100 (Alexander, 2017).

Executive function is a term describing a range of related higher order thinking skills that underpin the regulation of thoughts, emotions and behaviour – functions of great significance for goal achievement and everyday functioning (Jurado and Rosselli, 2007). Several different theories have addressed the structure of executive functioning, and the identified sub-components generally include: planning and organizing; working memory; inhibition; shifting; as well as initiating and monitoring (Diamond, 2013; Miyake and Friedman, 2012). Traditionally, executive functions have been measured either behaviourally, using executive function tasks, or via questionnaires to assess everyday executive function. The Behavioural Rating Inventory of Executive Function (BRIEF) is a much used questionnaire for assessing everyday executive function, and it is considered to provide ecologically valid measures of executive performance for children and adolescents across settings in their everyday life (Gioia et al., 2000).

There has been some interest in how executive functions relate to intelligence. In general populations, executive functions and intelligence are found to be related, but separate constructs (Friedman et al., 2006), and there are findings confirming that tests of executive functions assess something more than general intelligence (Friedman and Miyake, 2017). To obtain more knowledge of the relationship, it is important to also study everyday executive functions in populations with intelligence levels under the 2.5 percentile (Danielsson et al., 2012). Exploring the relationship between executive functions and intelligence in a population with mild intellectual disability, specifically, can have implications for diagnostical considerations and interventions for this group in clinical practice. If it turned out that executive functions were not strongly related to intelligence, it could indicate that interventions should build directly on strengths and difficulties in executive functions.

The relation between executive functions and intelligence has been studied in some such populations, for example, those on the autism spectrum who have lower IQ. Although results have often indicated a degree of executive difficulty in these populations, executive functions have not correlated highly with intelligence (Bertollo and Yerys, 2019; Merchán-Naranjoa et al., 2016). For instance, people with autism spectrum conditions can have intelligence scores within the average range, together with executive problems. Several studies have found executive difficulties in groups of people with autism spectrum conditions with mixed IQ levels (Craig et al., 2016; Garon et al., 2018; Hill, 2004; Roelofs et al., 2015; Weismer et al., 2018). Further, there are findings supporting syndrome-specific profiles of executive functions for individuals with Williams-, Downs and Prader-Willi syndrome (Carney et al., 2013; Chevalère et al., 2013, 2015; Costanzo et al., 2013; Csumitta et al., 2022; Daunhauer et al., 2014; Grieco et al., 2015; Hocking et al., 2015; Lee et al., 2011). The BRIEF has also been used to describe everyday executive functions across the early lifespan in groups with intellectual disability with Down syndrome and sex chromosome trisomy (XXX and XXY) (Lee et al., 2015; Loveall et al., 2017).

There is less knowledge about everyday executive functions, and how they relate to intelligence, in clinical groups of children and adolescents with mild intellectual disability and mixed aetiology, without concurrent autism spectrum condition. In one relevant study using behavioural measures, Danielsson et al. (2012) found a mixed picture of executive function ability in children with intellectual disabilities, with evidence for difficulties in sub-components such as inhibition, planning and non-verbal executive-loaded working memory, but equivalent performance in sub-components such as switching, verbal executive-loaded working memory and most fluency tasks, compared to mental-age matched peers. A further study using the BRIEF in a school setting, with special education teachers as respondents, reported that all mean scores of the children with intellectual disability differed significantly from the normative sample, indicating broad impairments in executive functioning, with lowest results on the Initiating and Working memory scales (Memisevic and Sinanovic, 2014). Previous studies of children and adolescents with mild intellectual disability has, to the best of our knowledge, not used everyday measures of executive functions with parents as respondents in this context.

It is not clear how everyday executive functions relate to adaptive behaviour in children and adolescents with mild intellectual disability. The executive function “inhibitory control”, measured behaviourally, has been found to be a significant developmental and modulation factor for the specific adaptive behaviours “conceptual and practical skills” in children and adolescents with mild intellectual disability (Gligorovic and Buha Ethurovic, 2014). The mild form of intellectual disability, with standardised scores on IQ and adaptive behavioural measures between 50 and 69, is the most common of the intellectual disabilities, and is also the form that has the highest rates of unexplained aetiology (Patel et al., 2020). Because of this, there is often little information available about what development to expect, and how to give the best developmental support, which in other developmental disorders, or syndromes, often comes with the diagnosis (Vasudevan and Suri, 2017).

Using a parent/caregiver-report, like the BRIEF to assess everyday executive functions in this group of children and adolescents can give information regarding strengths and difficulties, which can enable more targeted interventions that are specifically tailored according to the individuals´ needs and abilities. Gaining more knowledge regarding how everyday executive functions relate to intelligence and adaptive behaviour in children and adolescents with mild intellectual disability can be useful for clinicians wanting to provide the family and teachers with much needed information of what to expect, and where to place support in the life of children and adolescents with mild intellectual disability.

### Aims of the present study

The primary aim of the present study was to explore the relationship between everyday executive functions and intelligence in a group of children and adolescents with mild intellectual disability. A secondary aim was to explore whether intelligence or executive functions best would predict adaptive behaviour in this group. Previous research did not allow us to make specific hypotheses regarding the aims.

## Methods

### Procedure and Participants

**Figure 1.**
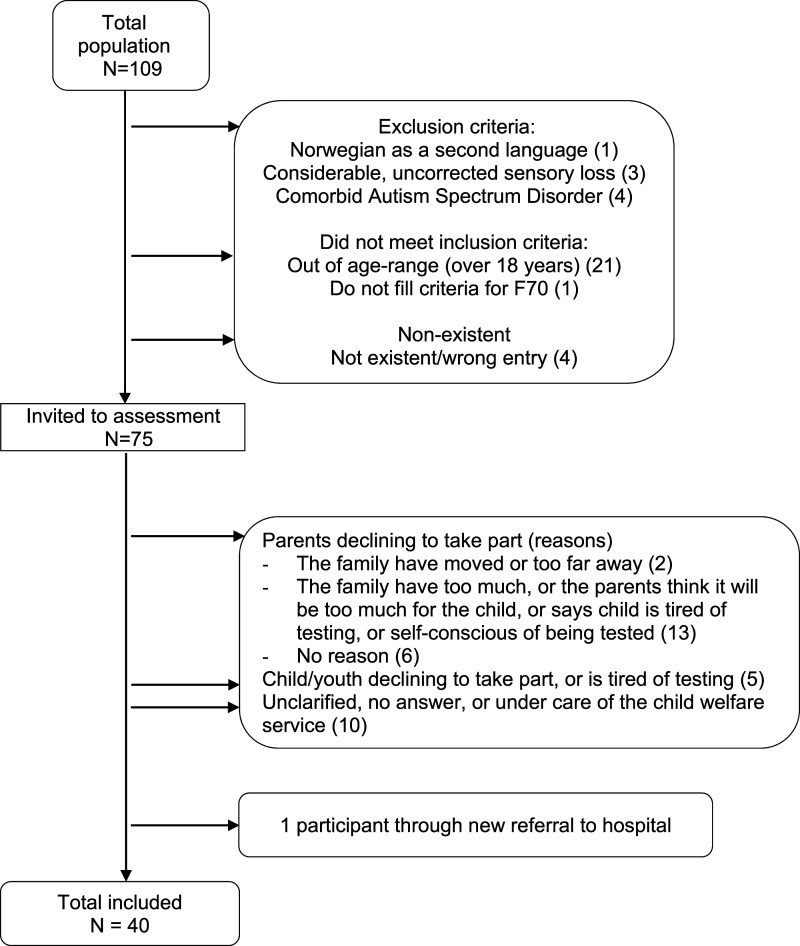
Flowchart illustrating subject recruitment and attrition

Initially, a total of 75 children and adolescents, previously diagnosed with mild intellectual disability from habilitation hospital outpatient clinics in the Central Norway Region, were invited to participate in the study, see Figure 1. Inclusion criteria were a chronological age between 10-18 years, and a diagnosis of mild intellectual disability. Exclusion criteria were having a comorbid diagnosis of autism spectrum disorder, not having Norwegian as a first language, and having large uncorrected sensory loss.

The final sample consisted of 40 children and adolescents (60% males) in the age range of 10-17 (Mean 14.8, SD 2.1) with a diagnosis of mild intellectual disability, and mixed aetiology who participated in the study from September 2018 to July 2020. See Table 1 for participants´ characteristics and results on assessments. Initial recruitment criteria of a diagnosis of mild intellectual disability involves an expectancy of finding IQ and Vineland scores in the range of 50-69. However, repeat assessments for the current study showed wider variability than this. Inclusion to the study was based on the original assessments and diagnostic considerations, acknowledging that diagnostic conclusions are complex, and based on comprehensive assessments. Because the repeat assessments were conducted for the purpose of the study, ensuring uniformity in administration and interpretation for the participants, they were used in the analyses.

**Table 1.** Descriptive statistics for the participants. Number of available observations (n), Mean (SD) and min-max except where otherwise specified.

Characteristics	n (%)	mean (SD)	min-max
Female sex	16 (40%)		
Age 10-13y 14-17y	40 11 (27.5%) 29 (72.5%)	14.8 (2.1)	10-17
Wechsler (FSIQ)	36	56.9 (11.1)	40-88
BRIEF (GEC)	37	68.5 (12.9)	40-90
Vineland (ABC)	36	64,3 (11.4)	44-92

Wechsler tests: Wechsler Intelligence Scale for Children Fourth Edition (WISC-IV) and Wechsler Adult Intelligence Scale Fourth Edition (WAIS-IV), Full IQ; BRIEF, Behaviour Rating Inventory of Executive Function, Global Executive Composite (GEC); Vineland ABS-II, Vineland Adaptive Behavior Scale Second Edition, Adaptive Behavior Composite (ABC)

## Materials and measures

*Adaptive behaviour:* To measure the youth´s adaptive behaviour, parents/caregivers were interviewed with the Vineland Adaptive Behavior Scales Interview, Second Edition, (Vineland ABS-II, n=36) (Sparrow et al., 2005; Sparrow et al., 2011). The Vineland ABS-II is a standardised, structured parent/caregiver interview and questionnaire of adaptive behaviour, with Scandinavian forms and norms, age 2-21 years. For the age-group 10-17 the scale is composed of the indexes Communication, Daily Living Skills and Socialization, which make up the Adaptive Behavior Composite (ABC), which is the measure used in this study. There is support for both the content validity and the structure of the scales, in addition to very satisfactory internal consistency and split-half reliability for both the ABC and the index scores (> 0.90) (Heyerdahl and Eikeseth, 2014).

*Everyday executive function:* To assess the children and adolescents´ everyday executive functions, parents or caregivers filled in the Behavior Rating Inventory of Executive Function (BRIEF, n=36) (Fallmyr and Egeland, 2011; Gioia et al., 2000). The BRIEF used in this study is a standardised parent report questionnaire for children and adolescents aged 5-18 years, and the Norwegian version used with American norms is reported as valid, with good psychometric properties (Sørensen and Hysing, 2014). A study has demonstrated that the BRIEF is valid, with sound psychometric properties, used in a group of school children with intellectual disability and Down syndrome (Esbensen et al., 2019). For the current study, The Global Executive Composite (GEC), a summary score that incorporates all the BRIEF clinical scales, was used as a measure of everyday executive functions. The direction of this scale is opposite compared to the other scales used in this study, i.e., higher GEC scores indicate more problems, and a T-score of 65 (i.e., one and a half standard deviation above the mean of 50), or more, indicates problems of clinical significance.

*Intelligence:* For participants where intelligence tests results were more than two years old, new tests were administrated (n=38), using the Wechsler Intelligence Scale for Children, Fourth Edition (WISC-IV, n=36), or the Wechsler Adult Intelligence Scale, Fourth Edition (WAIS-IV, n=2) (Wechsler, 2009; Wechsler, 2011). For the two participants with newer tests than two years, these WISC-IV results were used. The Wechsler tests were used to measure the full-scale intelligence quotient (FSIQ), both verbal and non-verbal, with index and full scores with a mean of 100, and SD=15. The psychometric analyses of the WISC-IV suggests that the Norwegian version replicates the American and Swedish versions concerning reliability (FSIQ *r*= 0.97), and also for correlations between subtests and indexes (Wechsler, 2009). For the Scandinavian WAIS-IV the average reliability coefficients (across age) for the four indexes were also high, varying from 0.90 to 0.94 (Lindau and Najström, 2019).

*Mild intellectual disability* was already diagnosed based on the ICD-10 criteria (World Health Organization, 2016) by psychologists and medical doctors in specialized outpatient clinics, following standardised procedures and requirements of significant limitations in intellectual functioning and in adaptive behaviour on well-validated intelligence batteries such as the Wechsler tests and assessment tools of adaptive behaviour such as the Vineland.

### Statistical analyses

Independent samples t-tests were conducted to examine whether scores on the BRIEF Global Executive Composite (GEC), Wechsler Full-Scale Intelligence Quotient (FSIQ), and Vineland-II Adaptive Behavior Composite (ABC) differed based on gender or age groups. The association between Wechsler Full-Scale Intelligence Quotient (FSIQ) and BRIEF Global Executive Composite (GEC) was analysed with the Pearson correlation coefficient. The correlated correlation coefficients were compared using the method based on the Fisher z-transformation, as recommended by Meng et al. (1992). A linear regression analysis was conducted with Vineland-II Adaptive Behavior Composite (ABC) as dependent variable, and Wechsler Full-Scale Intelligence Quotient (FSIQ) and BRIEF Global Executive Composite (GEC) as predictors, one at a time, and simultaneously. These analyses were done unadjusted, and adjusted for age and sex. Missing values were handled using available case analysis, that is, in each analysis, all cases with data on the relevant variables were included. Normality of residuals was confirmed by visual inspection of QQ-plots. Ninety-five percent confidence intervals are reported where relevant, and we regard two-sided *p*-values <0.05 to indicate statistical significance.

## Results

Table 1 shows gender, age distribution, Wechsler Full-Scale Intelligence Quotient (FSIQ), BRIEF Global Executive Composite (GEC) and Vineland-II Adaptive Behavior Composite (ABC) scores. Independent sample t-tests were conducted to see whether scores of BRIEF (GEC), Wechsler (FSIQ) and Vineland-II (ABC) differed based on gender or age groups. Results indicated no significant differences in BRIEF (GEC) based on gender (*t*(35)=-0.48, *p*=0.63, males: M=67.65, SD=12.63, females: M=69.79, SD=13.58), nor age-group (*t*(35)=0.30, *p*= 0.77, 10-13 years: M=69.50, SD=13.09, 14-17 years: M=68.07, SD=12.00). There were no significant differences in Vineland-II (ABC) based on gender (*t*(34)=-0.15, *p*=0.88, males: M=64.00, SD=9.96, females: M=64.60, SD=13.42) nor age-group (*t*(34)=-0.37, *p*=0.71, 10-13 years: M=63.100, SD=10.85, 14-17 years: 64.69, SD=11.71).

With respect to Wechsler (FSIQ), independent sample t-tests indicated a result that approached a significant difference *t*(34)=2.008, *p*=0.053 based on gender, where the males (M=59.73, SD=12.09) scored higher than the females (M=52.43, SD=7.71). Results indicated no significant differences in Wechsler (FSIQ) based on age-groups (*t*(34)=0.83, *p*=0.41), where the youngest group scored (M=59.56, SD=7.97) and the oldest group (M=56.00, SD=11.94).

### Correlation

There was a weak and non-significant correlation (*r*=-0.12, *p*=0.51) between the BRIEF (GEC) and the Wechsler (FSIQ). There was also a weak and non-significant correlation (*r*=0.21, *p*=0.23) between the Wechsler (FSIQ) and the Vineland-II (ABC), although this was of slightly higher magnitude. The correlation between the BRIEF (GEC) and the Vineland-II (ABC) was moderately strong and significant (*r*=-0.44, *p*=0.009). These two correlation coefficients (Wechsler (FSIQ) - Vineland-II (ABC) versus BRIEF (GEC) - Vineland-II (ABC)) were significantly different (*p*=0.007).

### Regression analysis

Results of linear regression analyses are shown in Table 2. Wechsler (FSIQ) was not a significant predictor of Vineland-II (ABC) (unstandardised regression coefficient 0.25, *p*=0.23), whereas the BRIEF (GEC) was a significant predictor (unstandardised regression coefficient -0.40, *p*=0.009). As seen in Table 2, the regression coefficients were similar in the analyses with one predictor at a time, and with both predictors in the model. Analyses adjusted for age and sex gave substantially the same results.

**Table 2.** Regression analyses with Adaptive Behaviour Composite as dependent variable, and IQ and BRIEF as predictors.

Predictors	N	Unstandardised regression coefficient	Confidence interval	*p*-value
FSIQ	33	0.25	-0.17 to 0.67	0.23
BRIEF	34	-0.40	-0.69 to -0.11	0.009
FSIQ and BRIEF	32			
FSIQ		0.26	-0.11 to 0.63	0.16
BRIEF		-0.42	-0.72 to -0.12	0.008

## Discussion

This study explored the role of everyday executive functions in relation to intelligence and adaptive behaviour in a group of children and adolescents with mild intellectual disability. Results indicated that intelligence and everyday executive functions were not significantly related, and that a global measure of everyday executive function was a significant predictor of adaptive behaviour in children and adolescents with mild intellectual disability. By contrast, full-scale IQ was not a significant predictor of adaptive behaviour in this group.

The finding that adaptive behaviour can be significantly predicted by everyday executive functions, but not by full-scale IQ, is important, because it confirms the structure of the existing diagnostic criteria in that we cannot predict how a child functions in everyday life based solely on an IQ measure (Bertelli et al., 2017). Further the results support earlier findings (Tassé et al., 2016), stating the importance of separately assessing both intelligence and adaptive behaviour, and weighing them equally in the process of diagnosing and understanding children and adolescents with intellectual disability. The findings indicate that higher order thinking skills, such as planning, working memory, shifting and inhibition have more impact on adaptive behaviour, than fundamental verbal and non-verbal reasoning and problem-solving abilities. This can provide for a more nuanced and optimistic perspective on development and everyday life for the children and adolescents in this group, considering that adaptive real-life behaviours have been described as more susceptible to environmental influence, than has intelligence (Sparrow et al., 2011). The findings show that there is a relationship between everyday executive functions and adaptive behaviours in this group, which suggests that support and help should be aimed at executive functions and adaptive behaviours. It would be of interest for the children and adolescents in this group that future research explores the underlying mechanisms further. Adaptive behaviours are acquired through everyday life experiences, and the findings in the present study are in line with research stating the importance of recognizing the adaptive skills acquired during the life-span, as described by Maulik et al. (2011).

The significance of adaptive behaviour should be acknowledged, and the strong relationship between everyday executive functions and adaptive behaviour should be recognized. Assessment of everyday executive abilities and adaptive behaviour can provide valuable information about individual strengths and difficulties in everyday settings, which again can create a basis for educational planning and developmental support. Shifting the focus, from levels of intelligence to strengths and difficulties across cognitive, executive and adaptive abilities, can lead to a more realistic picture of functioning in children and adolescents with mild intellectual disability. More focus on adaptive behaviours can contribute to the discovery of the individual´s resources.

Research in the population without intellectual disability has suggested that intelligence and executive functions are related, but separate constructs (Friedman et al., 2006). We wanted to study the relationship between intelligence and everyday executive functions in a group of children and adolescents with mild intellectual disability. Previous research using behavioural measures has reported a mixed picture of executive function ability in children with intellectual disabilities (Danielsson et al., 2012), while others, using the BRIEF teacher report forms to assess everyday executive functions have reported findings indicating broad impairments in executive functioning (Memisevic and Sinanovic, 2014). In the present study, even though significantly lower than the mean in the population without intellectual disability, everyday executive functions did not covary significantly with intelligence. This could suggest that earlier findings in populations without intellectual disability, indicating that the two constructs are separate, is also valid for this population. This means that executive abilities can vary across intellectual levels in a group of children and adolescents with mild intellectual disability. Hence, parent-reported everyday executive functions can give additional information concerning function across daily-life settings in children and adolescents with mild intellectual disability, that can be useful in optimizing developmental support and educational planning.

The current study shows that we cannot derive information about either executive function or adaptive ability from measures of intelligence, as these measures were not significantly related to each other. The implication is that we need to look at the broader aspects of functioning in obtaining a fuller understanding of children and adolescents with mild intellectual disability. Additionally, the prevalence of mental health problems has been found to be many times higher in groups of children and adolescents with intellectual disability, compared to children and adolescents without intellectual disability, and commonly reported conditions have been depressive disorders, conduct disorders and anxiety disorders (Buckley et al., 2020). It is possible that more knowledge concerning the role of executive functions, known to be important for planning and execution of behaviour, can add to our understanding of the increased prevalence of mental health problems in this group.

It could be argued that the correlation found between the global measure of executive function and adaptive behaviour comes from a possible overlap in what the two tools assess, or because they both are parent ratings, unlike the full-scale IQ measure from the Wechsler intelligence test, which is directly assessed. It could also be that the concepts themselves overlap. In the field of assessment of executive functions, intelligence and adaptive behaviour, there is the inevitable challenge associated with the purity of the definition of concepts associated with both the genotype and phenotype. Also relevant in this context, is the task-impurity problem (Miyake and Friedman, 2012), that points to the difficulty of purely testing one domain without relying, or getting noise from other areas of cognition. This is a methodological challenge in assessing executive functions in particular, although it is possible that using the global executive composite measure from the BRIEF, which combines sub-components of executive function, might have negated this difficulty to some extent.

In spite of these uncertainties, this study adds weight to the notion that because of great variation in cognitive profiles for people with intellectual disability, it is important to assess individual strengths and weaknesses in executive, and other specific cognitive functions in order to reach a better understanding of aspects concerning abilities of daily life (Bertelli et al., 2017), and to be able to plan effective interventions or educational programmes.

### Strengths and limitations

A possible limitation of the study was that the age distribution was somewhat skewed, with more children in the older age-groups, which could possibly impact on the results. Also, there were more males than females in the group. Analyses showed that there were no significant differences in intelligence, adaptive behaviour, or everyday executive functions based on gender or age-group. Knowing that mild intellectual disability was the main inclusion criterion, the skewness of age in the selection could reflect that mild intellectual disability is often diagnosed later in adolescence, rather than earlier in childhood. Another limitation is that new testing gave a wider range of IQ scores than expected on the basis of the diagnostic criteria of IQ scores in the 50-69 range (World Health Organization, 2016). As stated, the study kept to the originally identified sample because diagnosing mild intellectual disability is a complex process, where multiple factors are considered. Because one can expect variation in results on separate cognitive domains in this group, there is reason to believe that there has been careful clinical consideration of results in the different domains of cognitive and adaptive abilities to reach the diagnostic conclusions. The wider range of scores could also reflect developmental changes or test error. The strength of this study is that we have explored how everyday executive functions relate to both intelligence and adaptive behaviour, the corner-stones in diagnosing intellectual disability, in a clinical group that is not often the focus of attention in research.

## Conclusions

The results from the present study show that adaptive behaviour cannot be derived from knowing the IQ of children or adolescents with mild intellectual disability, as these constructs were not significantly related to each other. Further, there was no significant correlation between everyday executive function and intelligence. This offers support to findings in the population without intellectual disability, that intelligence and everyday executive functions are separate constructs. The key finding, that adaptive behaviour was predicted by everyday executive function, underpins the importance of collecting knowledge about function across settings, to obtain a richer and more nuanced understanding of functioning in children and adolescents with mild intellectual disability. Through assessing everyday executive abilities in the real-world setting, by use of a parent/caregiver-rated inventory such as the BRIEF, clinicians and families can acquire important information which again can provide a basis for optimizing support for children and adolescents with mild intellectual disability. Acknowledging the strong association between everyday executive functions and adaptive behaviour can lead to more targeted support for children and adolescents with mild intellectual disability.
